# Breast cancer risk factors in relation to estrogen receptor, progesterone receptor, insulin-like growth factor-1 receptor, and Ki67 expression in normal breast tissue

**DOI:** 10.1038/s41523-017-0041-7

**Published:** 2017-10-02

**Authors:** Hannah Oh, A. Heather Eliassen, Andrew H. Beck, Bernard Rosner, Stuart J. Schnitt, Laura C. Collins, James L. Connolly, Laleh Montaser-Kouhsari, Walter C. Willett, Rulla M. Tamimi

**Affiliations:** 1 Department of Epidemiology, Harvard T.H. Chan School of Public Health, Boston, MA USA; 2 Channing Division of Network Medicine, Brigham and Women’s Hospital and Harvard Medical School, Boston, MA USA; 3 Department of Nutrition, Harvard T.H. Chan School of Public Health, Boston, MA USA; 4 Section of Population Science, Rutgers Cancer Institute of New Jersey, New Brunswick, NJ USA; 5 Department of Pathology, Beth Israel Deaconess Medical Center, Harvard Medical School, Boston, MA USA; 6 PathAI, Cambridge, MA USA; 7 Department of Biostatistics, Harvard T.H. Chan School of Public Health, Boston, MA USA; 8 Department of Pathology, James H. Quillen College of Medicine, East Tennessee State University, Johnson City, TN USA

## Abstract

Studies have suggested that hormone receptor and Ki67 expression in normal breast tissue are associated with subsequent breast cancer risk. We examined the associations of breast cancer risk factors with estrogen receptor (ER), progesterone receptor (PR), insulin-like growth factor-1 receptor (IGF-1R), and Ki67 expression in normal breast tissue. This analysis included 388 women with benign breast disease (ages 17–67 years) in the Nurses’ Health Studies. Immunohistochemical staining was performed on tissue microarrays constructed from benign biopsies containing normal breast epithelium and scored as the percentage of epithelial cells that were positively stained. Ordinal logistic regression (outcomes in tertiles), adjusting for age and potential confounders, was performed to estimate odds ratios (OR) and 95% confidence intervals (CI) for the associations with risk factors. Alcohol consumption was positively associated (≥2.5 vs.<0.4 drink/wk: OR = 2.69, 95% CI = 1.26–5.75, *p*-trend = 0.008) and breastfeeding was inversely associated (≥6 months vs. never: OR = 0.11, 95% CI = 0.04–0.35, *p*-trend = 0.0003) with ER expression. Height (≥66 vs.<64 inches: OR = 2.50, 95% CI = 1.34–4.67, *p*-trend = 0.005) and BMI at age 18 (≥22 vs.<20 kg/m^2^: OR = 2.33, 95% CI = 1.18–4.62, *p*-trend = 0.01) were positively associated with PR expression. Body size at age 5–10 years was inversely associated with Ki67 (Level ≥ 2.5 vs. 1: OR = 0.55, 95% CI = 0.30–1.01, *p*-trend = 0.03). Premenopausal BMI (≥25 vs.<20 kg/m^2^) was positively associated with cytoplasmic IGF-1R (OR = 5.06, 95% CI = 1.17–21.8, *p*-trend = 0.04). Our data suggest that anthropometrics, breastfeeding, and alcohol intake may influence the molecular characteristics of normal breast tissue, elucidating the mechanisms by which these risk factors operate. However, larger studies are required to confirm these results.

## Risk factors: Alcohol, breastfeeding and body traits tied to molecular biomarkers

Body size, alcohol intake and breastfeeding may affect the molecular features of normal breast tissue to influence cancer risk. A team led by Hannah Oh from the Harvard T. H. Chan School of Public Health in Boston, USA, examined the link between behavioral and physiological risk factors for breast cancer and the expression levels of certain proteins (such as hormone receptors) that also promote cancer formation. By examining healthy breast tissue biopsied from 388 women diagnosed with benign breast diseases, the researchers showed that alcohol consumption, height and body mass index were all positively associated with the expression of various breast tissue markers, while breastfeeding and early-life body size were inversely associated. The findings point to a connection between lifestyle and breast tissue-specific molecular characteristics that underpin cancer risk.

## Introduction

Breast cancer is the most common cancer in women worldwide^1^. The majority of breast cancer cases occur among the women of age 50 years or older.^2,3^ Besides age, reproductive factors (e.g., nulliparity, older age at first birth),^4,5^ exogenous hormone use,^6,7^ height,^8^ and family history of breast cancer^9^ also increase the risk. Further, lifestyle factors such as alcohol consumption,^10^ lack of physical activity,^11^ and postmenopausal obesity^12^ are among the established risk factors for breast cancer. Although the exact mechanisms underlying these associations are not yet known, they are likely to involve hormone-dependent pathways as many of these factors are hormonally related.

Estrogen, progesterone, and insulin-like growth factor-1 (IGF-1), with their proliferative effects, are thought to be key hormones in breast cancer etiology.^13–15^ As proliferative effects of these hormones are largely mediated through their respective receptors,^16,17^ expression of these receptors in breast tissue may indicate tissue-specific responsiveness to these hormones. Ki67 is a cell proliferation marker; it is present only during active phases of the cell cycle.^18^ Highly proliferating cells, as reflected by high levels of Ki67, are prone to mutation initiation and expansion. Prior studies^19–22^ including ours^23,24^ have suggested that expression levels of estrogen receptor (ER), progesterone receptor (PR), IGF-1 receptor (IGF-1R), and Ki67 in cancer-free breast tissue may be associated with subsequent breast cancer risk.

Little is known about whether breast cancer risk factors influence molecular characteristics of normal breast before cancer develops. Previously, we have immunohistochemically (IHC) stained for ER, PR, IGF-1R, and Ki67 in benign breast biopsies within Nurses’ Health Study (NHS) and NHSII and examined the associations with subsequent breast cancer risk.^23,24^ Here, using the same biomarker data that were generated in the previous studies, we examined the associations of breast cancer risk factors with ER, PR, IGF-1R, and Ki67 expression in histopathologically normal breast epithelium among women with a diagnosis of benign breast disease (BBD) to help elucidate underlying mechanisms for these risk factors.

## Results

### Population characteristics

The mean age at benign breast biopsy was 45.1 years. The majority of women were premenopausal (70%) and parous (92%). Parous women, on average, had 3.1 pregnancies. Seventeen percent of women had proliferative lesions with atypical hyperplasia. Other demographic characteristics are shown in Table S1. Median percentages of ER-positive, PR-positive, and Ki67-positive cells in histopathologically normal breast tissue were 10, 6.7, and 4.2%, respectively, among these women with a previous diagnosis of BBD. Seventy percent of women had at least 1% membranous IGF-1R-positive cells and 28% had at least 1% cytoplasmic IGF-1R-positive cells.

Spearman correlations were modest between PR and ER (*r* = 0.34), and PR and membranous IGF-1R (*r* = 0.24) expression among both premenopausal and postmenopausal women (Table S2). In premenopausal women, cytoplasmic IGF-1R was positively correlated with ER (Spearman *r* = 0.22), PR (Spearman *r* = 0.23), and membranous IGF-1R (Spearman *r* = 0.29). In postmenopausal women, Ki67 was positively correlated with cytoplasmic IGF-1R (Spearman *r* = 0.36) but inversely correlated with membranous IGF-1R (Spearman *r* = −0.43) and PR (Spearman *r* = −0.34).

### Age

Older women (≥50 vs.<40 years) had slightly higher median levels of ER expression (10.9 vs. 9.6%; *p*-trend = 0.001) and lower median levels of Ki67 expression (2.7 vs. 4.9%; *p*-trend = 0.05) (Table 1). The associations did not change in the multivariable models (data not shown). Age was not associated with PR or IGF-1R expression.

**Table 1:** Associations of age with estrogen receptor (ER), progesterone receptor (PR), Ki67, and insulin-like growth factor-1 receptor (IGF-1R) expression levels in normal breast tissue among women with benign breast disease

	Age			Per 1-year OR^a^	*p*-trend^b^
	<40 years	40–49 years	≥50 years	(95% CI)	
Percentage of ER-positive cells					
*N*	22	78	58		
Median (IQR)	9.6 (2.3–15.5)	8.5 (4.0–14.7)	10.9 (8.0–18.3)	**1.07** (**1.03**–**1.11**)	**0.001**
Percentage of PR-positive cells					
*N*	42	100	73		
Median (IQR)	6.0 (2.7–11.9)	7.1 (4.2–11.7)	6.3 (2.0–11.4)	0.99 (0.97–1.02)	0.60
Percentage of Ki67-positive cells					
*N*	66	120	76		
Median (IQR)	4.9 (2.2–9.3)	4.5 (1.6–8.4)	2.7 (1.0–7.8)	**0.98** (**0.95–1.00**)	**0.05**
Percentage of IGF-1R-positive cells in membrane, *N* (%)					
<1.0 %	13 (26.5)	37 (32.5)	22 (26.8)		
1.0–32.9 %	11 (22.5)	30 (26.3)	20 (24.4)		
≥33.0 %	25 (51.0)	47 (41.2)	40 (48.8)	1.01 (0.98–1.03)	0.68
Percentage of IGF–1R-positive cells in cytoplasm, *N* (%)					
<1.0 %	38 (77.6)	83 (72.8)	56 (68.3)		
1.0–32.9 %	5 (10.2)	22 (19.3)	19 (23.2)		
≥33.0 %	6 (12.2)	9 (7.9)	7 (8.5)	1.02 (0.99–1.05)	0.30

*Note*: Values significant at the alpha level 0.05 are denoted in bold font

^a^ Odds ratios and 95% confidence intervals were estimated using ordinal logistic regression models on tertiles of marker expression ( < 7.3, 7.3–14.5, ≥ 14.6% ER-positive cells; < 4.0, 4.0–9.9, ≥ 10.0% PR-positive cells; < 2.3, 2.3–6.1, ≥ 6.2% Ki67-positive cells; < 1.0, 1.0–32.9, ≥ 33.0% membranous and cytoplasmic IGF-1R)

^b^ *P*-trend was estimated using the Wald test for a continuous age (years)

*ER* estrogen receptor, *PR* progesterone receptor, *IGF-1R* insulin-like growth factor-1 receptor, *OR* odds ratio, *CI* confidence interval, *IQR* interquartile range

### Anthropometric factors

Height (≥66 vs.<64 inches: multivariable [MV]-odds ratio [OR] = 2.50, 95% confidence interval [CI] = 1.34–4.67, *p*-trend = 0.005) and Body mass index (BMI) at age 18 (≥22 vs.<20 kg/m^2^: MV-OR = 2.33, 95% CI = 1.18–4.62, *p*-trend = 0.01) were positively associated with PR expression in normal breast tissue (Table 2). Height was also inversely associated with Ki67 expression when comparing women who were 64–65.9 vs. <64 inches tall (MV-OR = 0.50, 95% CI = 0.28–0.88); although not statistically significant, a similar magnitude of association was found when comparing women with height ≥66 vs. <64 inches (MV-OR = 0.60, 95% CI = 0.34–1.06). Body size at ages 5–10 years (level ≥ 2.5 vs. 1: MV-OR = 0.55, 95% CI = 0.30–1.01, *p*-trend = 0.03) was inversely associated with Ki67 expression. Similarly, BMI at age 18 (≥22 vs.<20 kg/m^2^) was inversely associated with Ki67 expression (MV-OR = 0.57, 95% CI = 0.31–1.02, *p*-trend = 0.06). Current BMI was positively associated with cytoplasmic IGF-1R among premenopausal women (≥25 vs.<20 kg/m^2^: MV-OR = 5.06, 95% CI = 1.17–21.8, *p*-trend = 0.04) (Table S3) but was not associated with other markers in normal breast tissue. None of the anthropometric factors were associated with ER expression.

**Table 2:** Associations of anthropometric factors with estrogen receptor (ER), progesterone receptor (PR), and Ki67 expression in normal breast tissue among women with benign breast disease

	ER expression			PR expression			Ki67 expression		
		Age-adj^a^	MV-adj^b^		Age-adj^a^	MV-adj^b^		Age-adj^a^	MV-adj^b^
	*N*	OR (95%CI)	OR (95%CI)	*N*	OR (95%CI)	OR (95%CI)	*N*	OR (95%CI)	OR (95%CI)
Height									
<64 inches	47	1.0 (ref)	1.0 (ref)	68	1.0 (ref)	1.0 (ref)	84	1.0 (ref)	1.0 (ref)
64–65.9 inches	47	0.62 (0.29, 1.33)	0.65 (0.30, 1.45)	63	1.68 (0.88, 3.19)	1.80 (0.93, 3.50)	86	**0.55** (**0.32, 0.97)**	**0.50** (**0.28, 0.88)**
≥66 inches	64	1.13 (0.56, 2.28)	1.24 (0.59, 2.60)	84	**2.27** (**1.25, 4.14)**	**2.50** (**1.34, 4.67)**	92	0.63 (0.37, 1.10)	0.60 (0.34, 1.06)
*p*-trend		0.51	0.41		**0.01**	**0.005**		0.17	0.14
Body size at ages 5–10 years									
Level 1	47	1.0 (ref)	1.0 (ref)	65	1.0 (ref)	1.0 (ref)	84	1.0 (ref)	1.0 (ref)
Level 1.5–2	49	0.87 (0.41, 1.85)	1.01 (0.45, 2.23)	66	1.14 (0.61, 2.15)	1.39 (0.72, 2.68)	74	1.26 (0.70, 2.26)	1.25 (0.68, 2.29)
Level ≥ 2.5	44	0.78 (0.36, 1.69)	0.71 (0.31, 1.64)	62	1.00 (0.52, 1.90)	1.05 (0.53, 2.06)	77	**0.55** (**0.31, 0.99)**	0.55 (0.30, 1.01)
*p*-trend		0.53	0.41		0.95	1.00		**0.03**	**0.03**
BMI at age 18 years									
<20 kg/m^2^	57	1.0 (ref)	1.0 (ref)	79	1.0 (ref)	1.0 (ref)	101	1.0 (ref)	1.0 (ref)
20–21.9 kg/m^2^	45	0.84 (0.41, 1.75)	1.01 (0.46, 2.22)	67	1.57 (0.86, 2.86)	**1.95** (**1.04, 3.64)**	80	0.83 (0.48, 1.42)	0.81 (0.47, 1.40)
≥22 kg/m^2^	41	1.02 (0.48, 2.17)	1.18 (0.54, 2.59)	52	**2.07** (**1.08, 3.98)**	**2.33** (**1.18, 4.62)**	64	0.59 (0.33, 1.06)	0.57 (0.31, 1.02)
*p*-trend		1.00	0.69		**0.02**	**0.01**		0.08	0.06
Current BMI among premenopausal women									
<20 kg/m^2^	21	1.0 (ref)	1.0 (ref)	25	1.0 (ref)	1.0 (ref)	38	1.0 (ref)	1.0 (ref)
20–24.9 kg/m^2^	58	0.84 (0.33, 2.14)	0.73 (0.26, 2.09)	81	1.41 (0.62, 3.22)	1.12 (0.46, 2.76)	106	1.37 (0.69, 2.71)	1.54 (0.75, 3.14)
≥25 kg/m^2^	23	0.51 (0.17, 1.54)	0.51 (0.13, 2.01)	32	1.57 (0.60, 4.13)	1.23 (0.40, 3.75)	47	0.68 (0.31, 1.49)	0.80 (0.32, 1.99)
*p*-trend		0.20	0.35		0.45	0.74		0.13	0.36
Current BMI among postmenopausal women									
<20 kg/m^2^	5	0.73 (0.11, 4.94)	1.56 (0.17, 14.3)	4	1.98 (0.28, 14.0)	4.06 (0.51, 32.1)	4	1.22 (0.18, 8.14)	0.73 (0.10, 5.18)
20–24.9 kg/m^2^	21	1.0 (ref)	1.0 (ref)	36	1.0 (ref)	1.0 (ref)	36	1.0 (ref)	1.0 (ref)
≥25 kg/m^2^	14	0.24 (0.05, 1.07)	0.31 (0.06, 1.58)	17	0.62 (0.20, 1.88)	0.76 (0.23, 2.50)	15	0.68 (0.21, 2.16)	0.62 (0.19, 2.06)
*p*-trend		0.10	0.12		0.28	0.33		0.47	0.54

*Note*: Odds ratios and 95% confidence intervals were estimated using ordinal logistic regression models on tertiles of marker expression (< 7.3, 7.3–14.5, ≥ 14.6% ER-positive cells; < 4.0, 4.0–9.9, ≥ 10.0% PR-positive cells; < 2.3, 2.3–6.1, ≥ 6.2% Ki67-positive cells). *P*-trend was estimated using the Wald test for continuous variables (category-specific median values). Values significant at the alpha level 0.05 are denoted in bold font

^a^ Adjusted for age (years)

^b^ Multivariable (MV)-adjusted models include age (years), parity (nulliparous, parous), breastfeeding (month, missing), alcohol (g/d), height (inch), and BMI at age 18 (kg/m^2^, missing). For current BMI among postmenopausal women, multivariable models adjusted for age (years), alcohol (g/d), and height (inch) only due to limited sample size

*ER* estrogen receptor, *PR* progesterone receptor, *OR* odds ratio, *CI* confidence interval, *BMI* body mass index

### Reproductive factors

Among parous women, total breastfeeding was inversely associated with ER expression (≥6 months vs. never: MV-OR = 0.11, 95% CI = 0.04–0.35, *p*-trend = 0.0003) (Table 3). Other reproductive factors were not associated with ER, PR, Ki67, or IGF-1R expression (Table 3; Table S3).

**Table 3:** Associations of reproductive factors with estrogen receptor (ER), progesterone receptor (PR), and Ki67 expression in normal breast tissue among women with benign breast disease

	ER expression			PR expression			Ki67 expression		
		Age-adj^a^	MV-adj^b^		Age-adj^a^	MV-adj^b^		Age-adj^a^	MV-adj^b^
	*N*	OR (95%CI)	OR (95%CI)	*N*	OR (95%CI)	OR (95%CI)	*N*	OR (95%CI)	OR (95%CI)
Age at menarche									
≤12 years	79	1.0 (ref)	1.0 (ref)	108	1.0 (ref)	1.0 (ref)	134	1.0 (ref)	1.0 (ref)
13 years	38	**0.45** (**0.22, 0.94)**	**0.41** (**0.19, 0.89)**	52	0.95 (0.52, 1.74)	0.82 (0.44, 1.54)	73	0.90 (0.53, 1.52)	0.86 (0.50, 1.47)
≥14 years	40	0.72 (0.35, 1.47)	0.68 (0.32, 1.45)	53	0.88 (0.48, 1.63)	0.79 (0.42, 1.49)	53	0.79 (0.44, 1.44)	0.74 (0.40, 1.37)
*p*-trend		0.23	0.20		0.69	0.43		0.44	0.32
Parity									
Nulliparous	12	1.0 (ref)	1.0 (ref)	19	1.0 (ref)	1.0 (ref)	22	1.0 (ref)	1.0 (ref)
1 birth	9	4.93 (0.89, 27.4)	5.06 (0.84, 30.4)	11	2.27 (0.57, 9.06)	2.20 (0.51, 9.39)	16	1.11 (0.34, 3.64)	1.13 (0.33, 3.92)
≥2 births	83	1.22 (0.39, 3.78)	2.16 (0.62, 7.56)	112	1.94 (0.77, 4.89)	2.24 (0.81, 6.16)	138	0.95 (0.40, 2.25)	1.00 (0.38, 2.59)
p-trend		0.86	0.38		0.20	0.14		0.85	0.94
Age at first birth (among parous women)									
<25 years	80	1.0 (ref)	1.0 (ref)	100	1.0 (ref)	1.0 (ref)	131	1.0 (ref)	1.0 (ref)
25–29 years	51	**0.50** (**0.25, 0.97)**	**0.47** (**0.23, 0.97)**	67	1.19 (0.67, 2.10)	1.20 (0.66, 2.17)	73	1.45 (0.85, 2.46)	1.57 (0.91, 2.70)
≥30 years	15	0.48 (0.17, 1.33)	0.59 (0.20, 1.76)	20	1.57 (0.64, 3.84)	1.71 (0.68, 4.31)	18	1.54 (0.62, 3.83)	1.78 (0.69, 4.55)
*p*-trend		0.08	0.21		0.30	0.24		0.19	0.10
Birth Index									
≤30	28	1.0 (ref)	1.0 (ref)	51	1.0 (ref)	1.0 (ref)	72	1.0 (Ref)	1.0 (ref)
31–59	39	0.78 (0.32, 1.93)	1.19 (0.44, 3.20)	56	1.15 (0.56, 2.36)	1.25 (0.58, 2.68)	72	0.89 (0.47, 1.69)	0.86 (0.44, 1.66)
≥60	47	0.67 (0.27, 1.68)	1.49 (0.50, 4.42)	56	0.66 (0.30, 1.42)	0.81 (0.34, 1.90)	69	1.14 (0.57, 2.29)	1.24 (0.60, 2.58)
*p*-trend		0.41	0.47		0.21	0.49		0.62	0.45
Total breastfeeding (among parous women)									
Never	35	1.0 (ref)	1.0 (ref)	47	1.0 (ref)	1.0 (ref)	53	1.0 (fef)	1.0 (ref)
<6 months	27	0.48 (0.18, 1.30)	0.41 (0.14, 1.17)	32	1.29 (0.56, 3.00)	1.04 (0.43, 2.51)	46	**0.45** (**0.22, 0.95)**	0.49 (0.23, 1.08)
≥6 months	26	**0.16** (**0.06, 0.46)**	**0.11** (**0.04, 0.35)**	41	0.81 (0.37, 1.77)	0.64 (0.28, 1.44)	52	0.54 (0.27, 1.11)	0.61 (0.29, 1.28)
*p*-trend		**0.001**	**0.0003**		0.41	0.21		0.34	0.53
Menopausal status									
Premenopausal	102	1.0 (ref)	1.0 (ref)	138	1.0 (ref)	1.0 (ref)	191	1.0 (ref)	1.0 (ref)
Postmenopausal	40	1.53 (0.63, 3.71)	1.91 (0.74, 4.96)	57	0.66 (0.30, 1.43)	0.70 (0.31, 1.56)	55	0.91 (0.44, 1.88)	0.96 (0.45, 2.07)
Age at menopause (among postmenopausal women)									
<50 years	17	1.0 (ref)	1.0 (ref)	27	1.0 (ref)	1.0 (ref)	27	1.0 (ref)	1.0 (ref)
≥50 years	13	0.55 (0.12, 2.57)	0.78 (0.16, 3.85)	22	0.35 (0.11, 1.07)	0.39 (0.12, 1.22)	20	1.36 (0.45, 4.13)	0.85 (0.25, 2.86)
*p*-trend		0.49	0.64		0.10	0.15		0.06	0.14

*Note*: Odds ratios and 95% confidence intervals were estimated using ordinal logistic regression models on tertiles of marker expression (< 7.3, 7.3–14.5, ≥ 14.6% ER-positive cells; < 4.0, 4.0–9.9, ≥ 10.0% PR-positive cells; < 2.3, 2.3–6.1, ≥ 6.2% Ki67-positive cells). *P*-trend was estimated using the Wald test for continuous variables (a continuous variable in years for age at menopause and category-specific median values for all other risk factors). Values significant at the alpha level 0.05 are denoted in bold font

^a^ Adjusted for age (years)

^b^ Multivariable (MV)-adjusted models include age (years), parity (nulliparous, parous), breastfeeding (month, missing), alcohol (g/d), height (inch), and BMI at age 18 (kg/m^2^, missing). For age at menopause, multivariable models adjusted for age (years), alcohol (g/d), and height (inch) only due to limited sample size

*ER* estrogen receptor, *PR* progesterone receptor, *OR* odds ratio, *CI* confidence interval

### Family history of breast cancer and other lifestyle factors

Higher levels of alcohol intake (≥2.5 vs.<0.4 drink/wk) was associated with higher levels of ER expression (MV-OR = 2.69, 95% CI = 1.26–5.75, *p*-trend = 0.008). Alcohol intake was also positively associated with PR expression in the age-adjusted models (OR = 2.03, 95% CI = 1.09–3.78, *p*-trend = 0.04) but the associations were attenuated after multivariable adjustment (MV-OR = 1.82, 95% CI = 0.97–3.44, *p*-trend = 0.08) (Table 4). Family history of breast cancer and physical activity were not associated with marker expression in normal breast tissue (Table 4; Table S3).

**Table 4:** Associations of family history of breast cancer and lifestyle factors with estrogen receptor (ER), progesterone receptor (PR), and Ki67 expression in normal breast tissue among women with benign breast disease

	ER expression			PR expression			Ki67 expression		
		Age-adj^a^	MV-adj^b^		Age-adj^a^	MV-adj^b^		Age-adj^a^	MV-adj^b^
	*N*	OR (95%CI)	OR (95%CI)	*N*	OR (95%CI)	OR (95%CI)	*N*	OR (95%CI)	OR (95%CI)
First-degree family history of breast cancer									
Absent	127	1.0 (ref)	1.0 (ref)	173	1.0 (ref)	1.0 (ref)	211	1.0 (ref)	1.0 (ref)
Present	31	1.28 (0.61, 2.71)	1.32 (0.61, 2.88)	42	0.97 (0.52, 1.81)	0.90 (0.47, 1.71)	51	0.80 (0.45, 1.40)	0.76 (0.43, 1.35)
Alcohol consumption									
<0.4 drink/wk	51	1.0 (ref)	1.0 (ref)	72	1.0 (ref)	1.0 (ref)	89	1.0 (ref)	1.0 (ref)
0.4 – 2.4 drink/wk	44	1.25 (0.59, 2.67)	1.29 (0.59, 2.84)	70	1.47 (0.80, 2.70)	1.40 (0.75, 2.62)	82	1.14 (0.65, 1.98)	1.17 (0.66, 2.05)
≥2.5 drink/wk	55	**2.47 (1.19, 5.11)**	**2.69 (1.26, 5.75)**	67	**2.03 (1.09, 3.78)**	1.82 (0.97, 3.44)	85	0.89 (0.51, 1.54)	0.97 (0.55, 1.69)
*p*-trend		**0.01**	**0.008**		**0.04**	0.08		0.55	0.77
Physical activity									
<5.5 MET-hr/wk	47	1.0 (ref)	1.0 (ref)	57	1.0 (ref)	1.0 (ref)	80	1.0 (ref)	1.0 (ref)
5.5 – 15.4 MET-hr/wk	38	0.81 (0.37, 1.81)	0.88 (0.37, 2.07)	60	0.83 (0.42, 1.61)	0.69 (0.34, 1.40)	74	1.04 (0.57, 1.87)	1.14 (0.62, 2.09)
≥15.5 MET-hr/wk	49	1.03 (0.49, 2.18)	1.33 (0.59, 2.96)	72	1.07 (0.57, 2.03)	1.06 (0.54, 2.09)	82	1.35 (0.76, 2.39)	1.52 (0.84, 2.74)
*p*-trend		0.83	0.41		0.66	0.56		0.27	0.15

*Note*: Odds ratios and 95% confidence intervals were estimated using ordinal logistic regression models on tertiles of marker expression (< 7.3, 7.3–14.5, ≥ 14.6% ER-positive cells; < 4.0, 4.0–9.9, ≥ 10.0% PR-positive cells; < 2.3, 2.3–6.1, ≥ 6.2% Ki67-positive cells). *P*-trend was estimated using the Wald test for continuous variables (category-specific median values). Values significant at the alpha level 0.05 are denoted in bold font

^a^ Adjusted for age (years).

^b^ Multivariable (MV)-adjusted models include age (years), parity (nulliparous, parous), breastfeeding (month, missing), alcohol (g/d), height (inch), and BMI at age 18 (kg/m^2^, missing)

*ER* estrogen receptor, *PR* progesterone receptor, *OR* odds ratio, *CI* confidence interval, *MET* metabolic equivalent task

Similar patterns of association were observed in sensitivity analyses after restricting the analyses to premenopausal women (Table S4) and after excluding cases diagnosed within 10 years since biopsy (data not shown).

## Discussion

In this analysis of normal breast tissue, we found evidence of associations between breast cancer risk factors and expression of tissue markers. Specifically, older women had higher ER and lower Ki67 expression in normal breast tissue. Anthropometric measures including height, body size at ages 5–10 years, and BMI at age 18 were associated with higher levels of PR and lower levels of Ki67 expression. Among premenopausal women, current BMI was associated with higher levels of cytoplasmic IGF-1R expression. Among parous women, breastfeeding was inversely associated with ER expression. Higher alcohol consumption was associated with higher levels of ER and PR expression. Our data suggest that these breast cancer risk factors may influence the molecular characteristics of normal breast tissue even before cancer develops.

Although previous studies were smaller in size, the distributions of marker expression we observed in the current study are comparable to those from previous studies of normal breast tissue.^25–30^ In study populations where the majority or all women were premenopausal, investigators have observed 3–7% ER-positive cells^25–27,29,30^, 12–29% PR-positive cells,^25–27^ and 3% Ki67-positive cells,^30^ in normal breast epithelium of cancer-free women. The estimates varied slightly across studies possibly due to differences in population characteristics (e.g., age, menopausal status, menstrual phase). In studies where menstrual phase information was available, ER expression was higher among women in follicular vs. luteal phase.^25,27,31–33^ Compared to premenopausal women, postmenopausal women had higher levels of ER (26–42%)^26,30^ and lower levels of Ki67 expression (0.34%).^30^ However, it is unknown whether expression levels of these markers in normal breast tissue from women with BBD are higher compared to those from women without BBD, as there is little data from women without BBD.

Consistent with findings from previous studies,^27,28,30^ we observed age was positively associated with ER but inversely associated with Ki67 expression in normal breast tissue. The inverse association between age and Ki67 expression is also consistent with physiologic changes women experience as they age. As women reach menopause, the ovaries stop producing estrogens and progesterone and their breasts undergo significant involution. In another study, some normal epithelium of involuting breasts was composed almost entirely of ER-positive cells,^30^ suggesting that ER-positive cells may be more slowly involuted than ER-negative cells. Although Ki67 was inversely associated with age, some studies suggested that the proportion of cells co-expressing ER and Ki67 may increase with age.^30^ In normal breast, ER-positive cells are unlikely to co-express Ki67^34^ and are thought to stimulate cell proliferation primarily in surrounding cells via paracrine activities;^34^ thus, high levels of ER and Ki67 co-expression may indicate the loss of control in cell division of ER-positive cells possibly due to high cumulative exposure to carcinogens.

Our findings of inverse associations between childhood body size and Ki67 expression are in line with the data from previous studies that have shown reduced risk of breast cancer^35–38^ and its intermediate markers (proliferative BBD, mammographic density)^39,40^ among women who were overweight and obese during early life. Studies have shown that women who were lean during their childhood have denser breasts^39^ and higher levels of circulating IGF-1^41^ in adulthood compared to those who were overweight or obese. These findings suggest that childhood body leanness may have long-term effects on breast tissue through these mechanisms that may be involved in cellular proliferation. Similarly, we also observed height, another anthropometric measure that correlates with early-life nutritional status and timing of puberty (e.g., fusion of the epiphyseal growth plates), was inversely associated with Ki67 expression. Furthermore, we did not observe an association between current BMI and Ki67 expression, which further supports the hypothesis that the early-life period, especially from menarche to the first childbirth, represents a window of susceptibility in which breast tissues are particularly susceptible to carcinogens.^42^

In this study, current BMI was not associated with ER and PR expression. While one study reported null findings,^27^ other studies have reported positive associations of current BMI with ER^32,43^ and PR expression.^32^ The difference in study findings may be due to the failure to take menstrual phase into account. Consistent with findings from a previous study,^27^ we also found positive associations of alcohol consumption with ER and PR expression, further supporting that alcohol may increase risk of breast cancer through hormonally-related mechanisms. Although previous studies have not found evidence of association with breastfeeding,^32^ we found strong inverse associations of breastfeeding with ER expression among parous women. However, little is known about the exact underlying mechanisms for these associations.

We acknowledge several limitations of this study. Given the small sample size, we had limited power to detect modest associations; however, to the best of our knowledge, the current study is the largest study of this topic. We performed multiple testing; thus, some of the positive findings could be due to chance. With the exploratory nature of this study, we did not take account of multiple testing in the analyses but have interpreted our results with caution. This hypothesis-generating study provides a basis for future study. Sampling variability in tissue microarray (TMA) coring may have contributed to measurement error in outcome. Furthermore, we do not have information on the phase of the menstrual cycle during which the benign biopsies were obtained. Studies have shown that levels of ER and Ki67 expression in normal breast tissue vary across cycle^25,31^ while PR levels do not.^31^ Lastly, our analyses were conducted among women with a previous diagnosis of BBD. Although the exact relationship is not clear, hormone receptor and Ki67 expression levels in normal terminal duct lobular units (TDLUs) among women who had benign breast biopsies may be different from those among healthy women who have never had a breast biopsy (e.g., field effect). Thus, our results may not be generalizable to women without BBD.

Despite these limitations, this study is the largest study to date to investigate the determinants of ER, PR, IGF-1R, and Ki67 expression levels in normal TDLUs. This study also included early-life factors such as BMI at age 18 and body size at ages 5–10 years. Furthermore, by using an automated image analysis system, we reduced observer error and measurement error in outcome.

In summary, breast cancer risk factors including age, alcohol consumption, breastfeeding, height, and early-life body size were associated with ER, PR, and Ki67 expression in normal breast tissue. These findings contribute to our understanding of breast cancer biology and suggest that these risk factors may influence the molecular characteristics of normal breast even before cancer develops. However, given our small sample size and lack of information on menstrual phase, further studies are required to confirm these results.

## Methods

### Study design and population

This analysis includes participants in the nested case-control study of breast cancer conducted among the subcohort of women who reported a diagnosis of biopsy-confirmed BBD in the NHS and NHSII cohorts. Details of this nested case-control study and the BBD assessment have been previously described.^24,44^ The NHS is an ongoing cohort study that began in 1976, including 121,700 female registered nurses aged 30–55 years. The NHSII is an ongoing cohort study that began in 1989, including 116,429 female registered nurses aged 25–42 years. In both cohorts, participants completed initial mailed, self-administered questionnaires that collected information on participants’ health behaviors, lifestyle factors, reproductive factors, and medical histories. Subsequent biennial follow-up questionnaires were used to assess updated information on a variety of known and suspected risk factors for chronic diseases, as well as newly diagnosed diseases including BBD confirmed by biopsy, and breast cancer. Cumulative response rates for both cohorts are > 90% and are similar among women with and without BBD diagnosis.

Cases were women with biopsy-confirmed BBD who reported a diagnosis of breast cancer during 1976–1998 for the NHS and 1989–1999 for the NHSII following their BBD diagnosis. When possible, four controls were selected for each case, matched on year of birth and year of benign breast biopsy, among women with biopsy-confirmed BBD who remained free of breast cancer at the time the matching case was diagnosed. We attempted to obtain BBD pathology records and archived biopsy specimens for all cases and controls from their hospital pathology departments; our ability to obtain biopsy blocks did not significantly differ by case and control status. Both cases and controls were included in the analysis because all tissue samples were collected prior to cancer diagnosis. To reduce potential reverse causation due to subclinical tissue change, women were excluded if they had evidence of in situ or invasive carcinoma at biopsy or reported a diagnosis of breast cancer within 6 months of their biopsy (*n* = 24). To assess the role of further reverse causation, we performed sensitivity analysis after excluding 53 women who developed breast cancer within 10 years since their benign biopsies.

This investigation was approved by the Institutional Review Board of the Brigham and Women’s Hospital. Completion of the self-administered questionnaire was presumed to imply informed consent.

### Risk factors assessment

Participants reported their risk factor information via questionnaires that were assessed prior to or around the time of biopsy. Body sizes at ages 5 and 10 years were recalled in 1988 (NHS) and 1989 (NHSII) using Stunkard’s nine-level pictogram (level 1: most lean; level 9: most overweight)^45^ as previously described.^37^ According to a validation study conducted within the Third Harvard Growth study, a longitudinal study of school children, recalled body size using this pictogram by women at ages 71–76 years has a good correlation with their measured BMI at ages 5 (Pearson *r* = 0.60) and 10 years (*r* = 0.65).^46^ Reported body sizes at ages 5 and 10 were averaged to represent childhood body size. Cumulative average of alcohol consumption was estimated by taking the average of alcohol consumption from age 18 to the years prior to benign biopsy. Cumulative average of physical activity was calculated by taking the average of physical activity (Metabolic Equivalent of Task [MET]-hr/wk) conducted during adulthood from enrollment in the cohort to the questionnaire cycle prior to benign biopsy. We also evaluated the associations with current levels of alcohol consumption and physical activity and found similar results. To examine the timing and spacing of births in relation to marker expression, we derived a birth index term as described previously.^47^ A higher birth index represents a higher number of births occurring at earlier ages. Height, BMI at age 18, birth index, alcohol consumption, and physical activity were categorized into tertiles.

### TMA construction and laboratory assays

Details of the TMA construction have been previously described.^24^ Briefly, six TMA blocks were constructed in the Dana Farber Harvard Cancer Center Tissue Microarray Core Facility (Boston, MA) by obtaining up to three 0.6-mm cores of histopathologically normal TDLUs from each formalin-fixed paraffin-embedded breast biopsy blocks. The “normal” TDLUs selected were regions of histopathologically normal tissue that were adjacent to benign lesions. Women were excluded if there was no breast tissue, or not enough tissue remaining on the block for coring.

For each IHC stain, a 5-μm paraffin section was cut from each TMA block and immunostained with its antibody (ERα: clone SP1, 1:40 diluation, Neomarkers, CA; PR: clone PgR 636, 1:150 diluation, Dako Corporation, CA; Ki67: clone SP6, 1:250 diluation, Vector Laboratories, CA; IGF-1R: clone 24–31, 1:100 diluation, Lab Vision, CA) after deparaffinization and rehydration. After blocking endogenous peroxidase activity, heat-induced epitope retrieval was performed on sections using the retrieval buffer (ERα: citrate pH 6.0 for 30 min; PR: citrate pH 6.0; Ki67/IGF-1R: EDTA pH 8.0 for 20 min) and then the antibodies were applied to the sections at room temperature. Appropriate positive and negative controls were included in all staining runs.

For ER, PR, and Ki67, immunostaining results of each core were interpreted using an automated computational image analysis system (Definiens Tissue Studio software, Munich, Germany) as previously described.^23^ For each stain, we used the Tissue Studio software to define an intensity and size threshold for nucleus identification and to define an intensity threshold for nuclear stain positivity. The automated analysis software was trained for scoring only the appropriate epithelial regions of the tissue. For each woman, we estimated the mean percentage of cells that were stain-positive across the cores, by weighting each core by its total cell count. The automated scoring data were moderately correlated with those manually scored by an expert pathologist (L.C.C.) on the subset of TMAs (73–327 cores) (Spearman *r* = 0.40–0.48).^23^ Because the data were skewed, we categorized into tertiles (ER: < 7.3%, 7.3–14.5%, ≥ 14.6%; PR: < 4.0%, 4.0–9.9%, ≥ 10.0%; Ki67: < 2.3%, 2.3–6.1%, ≥ 6.2%).

For IGF-1R, immunostaining results of each core were manually scored on a 0–5 scale according to the proportion of stain-positive cells (0%, 0.1–0.9%, 1.0–9.9%, 10.0–32.9%, 33.0–66.9%, ≥67.0%)^48^ for cytoplasmic and membranous staining separately by an expert pathologist (L.C.C.). Because our previous study^24^ suggested that the associations between IGF-1R expression and breast cancer risk may vary by cytoplasmic vs. membranous staining, we examined cytoplasmic and membranous staining separately. Further, cytoplasmic IGF-1R is thought to indicate internalized IGF-1R levels after IGF-1 has bound to it for IGF signaling.^49,50^ For each woman, we estimated the mean score across the cores and collapsed into three categories (<1.0%, 1.0–32.9%, ≥33.0%).

A total of 388 women (331 from the NHS and 57 from the NHSII; 82 cases and 306 controls) with at least one core of histopathologically normal TDLUs with evaluable staining were included in this study. Because this study was a secondary data analysis of markers that were initially stained for previous studies^23,24^ with varying grant aims and funding support, different numbers of TMAs were stained for each marker (4 TMAs for ER, 5 TMAs for PR and IGF-1R, 6 TMAs for Ki67) resulting in varying numbers of women with evaluable stainig. Among these 388 women, 158 women for ER, 215 women for PR, 245 women for IGF-1R, and 262 women for Ki67 were included in the analyses.

### Statistical analysis

In each risk factor analysis, we excluded particiants if they were missing age (an important confounder), exposure (risk factors), or outcome (tissue markers) data. Ordinal logistic regression was performed on ordinal categories of tissue marker expression (tertiles for ER, PR, and Ki67; three categories for IGF-1R) to estimate ORs and 95% CIs for the associations between the markers and the risk factors, adjusting for age. The ORs from the ordinal logistic regression indicates the odds of increasing one ordinal marker expression category (e.g., from the first to the second tertiles of ER expression or from the second to the third terile of ER expression) associated with risk factors (e.g., height ≥ 66 vs. <64 inches). We tested for model assumptions (proportional odds) using chi-square score test and did not find evidence for violation. In multivariable models, we also adjusted for potential confounders (parity, breastfeeding, alcohol intake, height, BMI at age 18; with missing indicators if necessary). Additional adjustment for menopausal status, current BMI (or change in BMI since age 18), age at menarche, and age at first birth did not change the results thus were not included in the final models. We performed a test for trend using category-speciic median values. Because expression levels of tissue markers may vary by menopausal status, we restricted our analyses to 272 women who were premenopausal at BBD biopsy (*n* = 102 ER, 138 PR, 165 IGF-1R, 191 Ki67) in sensitivity analyses. All statistical tests were two-sided with 5% type I error. Analyses were conducted with SAS version 9 (SAS Institute).

### Data availability

The data that support the findings of this study are available from the Nurses’ Health Studies but restrictions apply to the availability of these data, and so they are not publicly available. However, data are available from the authors upon reasonable request and with permission of Nurses’ Health Studies External Advisory Board. Additional data sharing information and policy details can be accessed at http://www.nurseshealthstudy.org/researchers.

## Electronic supplementary material

Supplementary Table 1-4
