# Supplementary material for: Breast cancer risk factors in relation to estrogen receptor, progesterone receptor, insulin-like growth factor-1 receptor, and Ki67 expression in normal breast tissue
Source: NPJ Breast Cancer. 2017 Oct 2;3:39. doi: 10.1038/s41523-017-0041-7 (PMC5624935; doi:10.1038/s41523-017-0041-7)
Supplement: Supplementary file 1 — Supplementary Table 1-4 [file 41523_2017_41_MOESM1_ESM.docx]

**Table S1. Characteristics of the study population at benign breast biopsy in the Nurses’ Health Study (NHS) and NHSII (N=388)**

|  | **Mean (SD) or N (%)** |
| --- | --- |
| Age, years | 45.1 (9.2) |
| Case-control status |  |
| - Case, % | 82 (21.1) |
| - Control, % | 306 (78.9) |
| Type of benign lesion |  |
| - Non-proliferative, % | 107 (27.6) |
| - Proliferative without atypia, % | 215 (55.4) |
| - Proliferative with atypical hyperplasia, % | 66 (17.0) |
| Height, inches | 64.6 (2.5) |
| Average body size at ages 5-10 years^1^ |  |
| - Level 1 (most lean), % | 125 (36.2) |
| - Level 1.5-2, % | 105 (30.4) |
| - Level 2.5-3, % | 48 (13.9) |
| - Level 3.5-4, % | 40 (11.6) |
| - Level ≥4.5 (most overweight), % | 27 (7.8) |
| BMI at age 18 years, kg/m^2^ | 21.0 (2.9) |
| BMI at biopsy, kg/m^2^ | 22.9 (5.8) |
| Age at menarche, years | 12.5 (1.4) |
| Parous, % | 357 (92.0) |
| Parity (among parous women) | 3.1 (1.6) |
| Age at first birth (among parous women) | 24.8 (3.2) |
| Menopausal status |  |
| - Premenopausal, % | 272 (70.1) |
| - Postmenopausal, % | 87 (22.4) |
| - Unknown, % | 29 (7.5) |
| Age at menopause (among postmenopausal women) | 47.9 (5.4) |
| Ever oral contraceptive use, % | 191 (49.6) |
| Ever smokers, % | 206 (53.5) |
| First-degree family history of breast cancer, % | 75 (19.3) |
| Cumulative average lifetime alcohol consumption ^1^, g/d | 4.70 (6.7) |
| Cumulative average adult physical activity ^2^, MET-hr/wk | 15.4 (17.9) |

^1^ Cumulative average of alcohol consumption starting at age 18 years to the years prior to benign biopsy.

^2^ Cumulative average of physical activity practiced during adulthood since enrollment in the cohort (1976 for the NHS and 1989 for the NHSII) to the years prior to benign biopsy. MET-hr/wk of total activity was estimated by multiplying the number of hr/wk of each activity with its corresponding average MET values (strenuous activity = 7 METs, moderate activity = 4.5 METs, walking = 3 METs) and summing the values from all activities.

Abbreviations: SD=standard deviation, BMI=body mass index, kg=kilogram, g=gram, MET-hr/wk=metabolic equivalent of task-hour/week.

**Table S2. Spearman correlations among tissue markers in normal breast tissue**

| **All women** | | | | | |
| --- | --- | --- | --- | --- | --- |
|  | **ER** | **PR** | **Ki67** | **Membranous IGF-1R** | **Cytoplasmic IGF-1R** |
| **ER** | 1.0 | 0.34*  (n=133) | -0.02  (n=99) | 0.05  (n=145) | 0.19*  (n=145) |
| **PR** |  | 1.0 | -0.12  (n=142) | 0.24*  (n=184) | 0.13  (n=183) |
| **Ki67** |  |  | 1.0 | -0.26*  (n=139) | -0.02  (n=138) |
| **Membranous IGF-1R** |  |  |  | 1.0 | 0.27*  (n=245) |
| **Cytoplasmic IGF-1R** |  |  |  |  | 1.0 |
| **Premenopausal women** | | | | | |
|  | **ER** | **PR** | **Ki67** | **Membranous IGF-1R** | **Cytoplasmic IGF-1R** |
| **ER** | 1.0 | 0.43*  (n=83) | 0.10  (n=68) | 0.09  (n=95) | 0.22*  (n=95) |
| **PR** |  | 1.0 | -0.03  (n=94) | 0.24*  (n=122) | 0.23*  (n=122) |
| **Ki67** |  |  | 1.0 | -0.18  (n=95) | -0.10  (n=95) |
| **Membranous IGF-1R** |  |  |  | 1.0 | 0.29*  (n=165) |
| **Cytoplasmic IGF-1R** |  |  |  |  | 1.0 |
| **Postmenopausal women** | | | | | |
|  | **ER** | **PR** | **Ki67** | **Membranous IGF-1R** | **Cytoplasmic IGF-1R** |
| **ER** | 1.0 | 0.35*  (n=35) | -0.13  (n=23) | 0.001  (n=34) | 0.06  (n=34) |
| **PR** |  | 1.0 | -0.34*  (n=38) | 0.32*  (n=44) | -0.13  (n=43) |
| **Ki67** |  |  | 1.0 | -0.43*  (n=34) | 0.36*  (n=33) |
| **Membranous IGF-1R** |  |  |  | 1.0 | 0.18  (n=59) |
| **Cytoplasmic IGF-1R** |  |  |  |  | 1.0 |

Abbreviations: ER=estrogen receptor; PR=progesterone receptor, IGF-1R=insulin-like growth factor-1 receptor.

* indicates p<0.05

**Table S3. Adjusted odds ratios (OR) and 95% confidence interval (CI) for associations between breast cancer risk factors and insulin-like growth factor-1 (IGF-1R) expression in normal breast tissue among women with benign breast disease**

|  | **Membranous IGF-1R** | | |  | **Cytoplasmic IGF-1R** | |
| --- | --- | --- | --- | --- | --- | --- |
|  | **N** | **Age-adj ^1^**  **OR (95% CI)** | **MV-adj ^2^**  **OR (95% CI)** | **N** | **Age-adj ^1^**  **OR (95% CI)** | **MV-adj ^2^**  **OR (95% CI)** |
| **Height** |  |  |  |  |  |  |
| <64 inches | 77 | 1.0 (ref) | 1.0 (ref) | 77 | 1.0 (ref) | 1.0 (ref) |
| 64-65.9 inches | 71 | 1.28 (0.70, 2.33) | 1.26 (0.68, 2.34) | 71 | 0.93 (0.45, 1.94) | 0.90 (0.43, 1.91) |
| ≥66 inches | 97 | 1.35 (0.78, 2.36) | 1.29 (0.72, 2.30) | 97 | 1.21 (0.63, 2.34) | 1.25 (0.63, 2.48) |
| p-trend |  | 0.31 | 0.43 |  | 0.51 | 0.45 |
| **Body size at ages 5-10 years** | | | | | | |
| Level 1 | 81 | 1.0 (ref) | 1.0 (ref) | 81 | 1.0 (ref) | 1.0 (ref) |
| Level 1.5-2 | 68 | 1.25 (0.69, 2.26) | 1.35 (0.72, 2.52) | 68 | 1.68 (0.83, 3.38) | 1.73 (0.83, 3.61) |
| Level ≥2.5 | 70 | 1.33 (0.73, 2.42) | 1.21 (0.65, 2.25) | 70 | 1.11 (0.53, 2.32) | 1.04 (0.49, 2.24) |
| p-trend |  | 0.37 | 0.58 |  | 0.87 | 0.97 |
| **BMI at age 18 years** |  |  |  |  |  |  |
| <20 kg/m^2^ | 97 | 1.0 (ref) | 1.0 (ref) | 97 | 1.0 (ref) | 1.0 (ref) |
| 20-21.9 kg/m^2^ | 75 | 0.91 (0.52, 1.59) | 0.96 (0.54, 1.69) | 75 | 0.83 (0.42, 1.64) | 0.86 (0.43, 1.73) |
| ≥22 kg/m^2^ | 55 | 1.63 (0.87, 3.05) | 1.54 (0.80, 2.95) | 55 | 1.29 (0.63, 2.61) | 1.20 (0.57, 2.49) |
| p-trend |  | 0.19 | 0.26 |  | 0.56 | 0.69 |
| **Current BMI among premenopausal women** | | | | | | |
| <20 kg/m^2^ | 28 | 1.0 (ref) | 1.0 (ref) | 28 | 1.0 (ref) | 1.0 (ref) |
| 20-24.9 kg/m^2^ | 102 | 2.19 (1.00, 4.80) | 2.25 (0.97, 5.23) | 102 | 2.17 (0.68, 6.96) | 2.81 (0.78, 10.1) |
| ≥25 kg/m^2^ | 35 | 2.15 (0.84, 5.49) | 1.75 (0.62, 4.99) | 35 | 3.56 (1.00, 12.7) | **5.06 (1.17, 21.8)** |
| p-trend |  | 0.24 | 0.62 |  | **0.05** | **0.04** |
| **Current BMI among postmenopausal women** | | | | | | |
| <20 kg/m^2^ | 7 | 0.86 (0.19, 3.91) | 1.20 (0.23, 6.35) | 7 | 3.87 (0.76, 19.8) | 4.03 (0.65, 25.0) |
| 20-24.9 kg/m^2^ | 37 | 1.0 (ref) | 1.0 (ref) | 37 | 1.0 (ref) | 1.0 (ref) |
| ≥25 kg/m^2^ | 15 | 2.60 (0.78, 8.63) | 2.96 (0.85, 10.4) | 15 | 1.10 (0.28, 4.42) | 1.03 (0.25, 4.27) |
| p-trend |  | 0.11 | 0.12 |  | 0.47 | 0.42 |
| **Age at menarche** |  |  |  |  |  |  |
| ≤12 years | 126 | 1.0 (ref) | 1.0 (ref) | 126 | 1.0 (ref) | 1.0 (ref) |
| 13 years | 64 | 1.47 (0.84, 2.59) | 1.47 (0.82, 2.61) | 64 | 0.80 (0.40, 1.60) | 0.76 (0.38, 1.54) |
| ≥14 years | 53 | 1.44 (0.78, 2.65) | 1.39 (0.74, 2.63) | 53 | 1.16 (0.58, 2.32) | 1.10 (0.53, 2.26) |
| p-trend |  | 0.16 | 0.21 |  | 0.82 | 0.95 |
| **Parity** |  |  |  |  |  |  |
| Nulliparous | 21 | 1.0 (ref) | 1.0 (ref) | 21 | 1.0 (ref) | 1.0 (ref) |
| 1 birth | 12 | 1.15 (0.30, 4.36) | 1.18 (0.30, 4.55) | 12 | **4.98 (1.07, 23.2)** | **5.73 (1.20, 27.5)** |
| ≥2 births | 134 | 1.45 (0.59-3.54) | 1.26 (0.49, 3.25) | 134 | 2.12 (0.64, 7.00) | 2.08 (0.59, 7.30) |
| p-trend |  | 0.40 | 0.64 |  | 0.40 | 0.48 |
| **Age at first birth (among parous women)** | | | | | | |
| <25 years | 119 | 1.0 (ref) | 1.0 (ref) | 119 | 1.0 (ref) | 1.0 (ref) |
| 25-29 years | 74 | 1.00 (0.58, 1.71) | 1.00 (0.57, 1.76) | 74 | 0.80 (0.42, 1.52) | 0.85 (0.44, 1.66) |
| ≥30 years | 20 | 1.94 (0.76, 4.92) | 2.08 (0.78, 5.52) | 20 | 1.04 (0.38, 2.85) | 1.26 (0.45, 3.55) |
| p-trend |  | 0.22 | 0.19 |  | 0.88 | 0.83 |
| **Birth Index** |  |  |  |  |  |  |
| ≤30 | 58 | 1.0 (ref) | 1.0 (ref) | 58 | 1.0 (ref) | 1.0 (ref) |
| 31-59 | 60 | 0.96 (0.47, 1.95) | 1.08 (0.52, 2.24) | 60 | 1.54 (0.64, 3.69) | 1.82 (0.73, 4.54) |
| ≥60 | 67 | 0.72 (0.34, 1.51) | 0.94 (0.42, 2.13) | 67 | 1.02 (0.39, 2.65) | 1.57 (0.53, 4.64) |
| p-trend |  | 0.34 | 0.84 |  | 0.79 | 0.55 |
| **Total breastfeeding (among parous women)** | | | | | | |
| Never | 53 | 1.0 (ref) | 1.0 (ref) | 53 | 1.0 (ref) | 1.0 (ref) |
| <6 months | 46 | 1.92 (0.88, 4.15) | 1.83 (0.81, 4.13) | 46 | 0.42 (0.17, 1.02) | **0.35 (0.13, 0.89)** |
| ≥6 months | 42 | 2.08 (0.95, 4.54) | 2.01 (0.89, 4.54) | 42 | 0.92 (0.41, 2.07) | 0.81 (0.35, 1.87) |
| p-trend |  | 0.17 | 0.21 |  | 0.61 | 0.66 |
| **Menopausal status** |  |  |  |  |  |  |
| Premenopausal | 165 | 1.0 (ref) | 1.0 (ref) | 165 | 1.0 (ref) | 1.0 (ref) |
| Postmenopausal | 59 | 0.89 (0.44, 1.83) | 1.09 (0.51, 2.30) | 59 | 0.78 (0.33, 1.85) | 0.89 (0.36, 2.21) |
| **Age at menopause (among postmenopausal women)** | | | | | | |
| <50 years | 25 | 1.0 (ref) | 1.0 (ref) | 25 | 1.0 (ref) | 1.0 (ref) |
| ≥50 years | 21 | 0.70 (0.23, 2.20) | 0.94 (0.27, 3.29) | 21 | 0.80 (0.20, 3.26) | 0.69 (0.15, 3.18) |
| p-trend |  | **0.04** | 0.07 |  | 0.78 | 0.89 |
| **First-degree family history of breast cancer** | | | | | | |
| Absent | 195 | 1.0 (ref) | 1.0 (ref) | 195 | 1.0 (ref) | 1.0 (ref) |
| Present | 50 | 0.68 (0.38, 1.22) | 0.73 (0.40, 1.32) | 50 | 1.22 (0.62, 2.40) | 1.22 (0.61, 2.46) |
| **Alcohol consumption** | | | | | | |
| <0.4 drink/wk | 74 | 1.0 (ref) | 1.0 (ref) | 74 | 1.0 (ref) | 1.0 (ref) |
| 0.4 – 2.4 drink/wk | 75 | **1.87 (1.02, 3.41)** | 1.86 (1.00, 3.45) | 75 | 1.36 (0.68, 2.71) | 1.26 (0.63, 2.55) |
| ≥2.5 drink/wk | 88 | 1.72 (0.97, 3.06) | 1.65 (0.92, 2.98) | 88 | 0.77 (0.38, 1.57) | 0.69 (0.34, 1.41) |
| p-trend |  | 0.19 | 0.25 |  | 0.26 | 0.16 |
| **Physical activity** |  |  |  |  |  |  |
| <5.5 MET-hr/wk | 70 | 1.0 (ref) | 1.0 (ref) | 70 | 1.0 (ref) | 1.0 (ref) |
| 5.5 – 15.4 MET-hr/wk | 65 | **1.98 (1.04, 3.76)** | 1.71 (0.88, 3.31) | 65 | 1.65 (0.78, 3.51) | 1.64 (0.75, 3.60) |
| ≥15.5 MET-hr/wk | 77 | 1.16 (0.64, 2.11) | 0.99 (0.52, 1.87) | 77 | 1.54 (0.75, 3.19) | 1.39 (0.64, 2.99) |
| p-trend |  | 0.99 | 0.66 |  | 0.34 | 0.56 |

Note: Odds ratios and 95% confidence intervals were estimated using ordinal logistic regression models for IGF-1R categories (<1.0, 1.0-32.9, ≥33.0% IGF-1R-positive cells). P-trend was estimated using the Wald test for continuous variables (a continuous variable in years for age at menopause and category-specific median values for all other risk factors).

^1^ Adjusted for age (years)

^2^ Adjusted for age (years), parity (nulliparous, parous), breastfeeding (month, missing), alcohol (drink/wk), height (inch), and BMI at age 18 (kg/m^2^, missing). For postmenopausal current BMI and age at menopause, multivariable models adjusted for age (years), alcohol (g/d), and height (inch) only due to limited sample size.

Abbreviations: IGF-1R=insulin-like growth factor-1 receptor, OR=odds ratio, CI=confidence interval, BMI=body mass index, g/d=gram/day, MET-hr/wk=metabolic equivalent of task-hours/week

**Table S4. Age-adjusted odds ratios (OR) and 95% confidence interval (CI) for associations of breast cancer risk factors with estrogen receptor (ER), progesterone receptor (PR), insulin-like growth factor-1 (IGF-1R), and Ki67 expression in normal breast tissue among PREMENOPAUSAL women with benign breast disease**

|  | **ER expression** | | | **PR expression** | | **Ki67 expression** | |  | **Membranous**  **IGF-1R expression** | **Cytoplasmic**  **IGF-1R expression** |  |  |  |
| --- | --- | --- | --- | --- | --- | --- | --- | --- | --- | --- | --- | --- | --- |
|  | **N** | | **OR (95%CI)** | **N** | **OR (95%CI)** | **N** | **OR (95%CI)** | **N** | **OR (95%CI)** | **OR (95%CI)** |  |  |  |
| **Height** |  | |  |  |  |  |  |  |  |  |  |  |  |
| <64 inches | 29 | | 1.0 (ref) | 39 | 1.0 (ref) | 61 | 1.0 (ref) | 51 | 1.0 (ref) | 1.0 (ref) |  |  |  |
| 64-65.9 inches | 35 | | 0.61 (0.24, 1.53) | 49 | 1.66 (0.76, 3.63) | 68 | 0.72 (0.38, 1.36) | 53 | 1.60 (0.78, 3.30) | 1.01 (0.41, 2.51) |  |  |  |
| ≥66 inches | 38 | | 1.00 (0.41, 2.43) | 50 | 2.02 (0.93, 4.40) | 62 | 0.94 (0.49, 1.80) | 61 | 1.09 (0.55, 2.18) | 1.54 (0.67, 3.56) |  |  |  |
| p-trend |  | | 0.80 |  | 0.10 |  | 0.97 |  | 0.97 | 0.27 |  |  |  |
| **Body size at ages 5-10 years** | | | |  |  |  |  |  |  |  |  | |  |
| Level 1 | 26 | | 1.0 (ref) | 36 | 1.0 (ref) | 50 | 1.0 (ref) | 50 | 1.0 (ref) | 1.0 (ref) |  |  |  |
| Level 1.5-2 | 34 | | 1.06 (0.41, 2.73) | 46 | 1.35 (0.61, 3.02) | 57 | 1.43 (0.71, 2.90) | 49 | 1.61 (0.78, 3.35) | 1.54 (0.64, 3.70) |  |  |  |
| Level ≥2.5 | 30 | | 0.90 (0.34, 2.38) | 44 | 1.01 (0.45, 2.28) | 63 | 0.60 (0.30, 1.20) | 51 | **2.42 (1.15, 5.09)** | 1.18 (0.48, 2.92) |  |  |  |
| p-trend |  | | 0.80 |  | 0.91 |  | 0.07 |  | **0.02** | 0.80 |  |  |  |
| **BMI at age 18 years** | | |  |  |  |  |  |  |  |  |  | |  |
| <20 kg/m^2^ | 38 | | 1.0 (ref) | 52 | 1.0 (ref) | 74 | 1.0 (ref) | 68 | 1.0 (ref) | 1.0 (ref) |  |  |  |
| 20-21.9 kg/m^2^ | 26 | | 1.10 (0.44, 2.79) | 40 | 2.06 (0.94, 4.51) | 54 | 0.93 (0.49, 1.78) | 46 | 1.53 (0.76, 3.10) | 0.91 (0.38, 2.18) |  |  |  |
| ≥22 kg/m^2^ | 31 | | 1.11 (0.46, 2.67) | 39 | **2.21 (1.01, 4.81)** | 53 | 0.59 (0.30, 1.13) | 43 | 1.71 (0.83, 3.51) | 1.27 (0.55, 2.92) |  |  |  |
| p-trend |  | | 0.81 |  | **0.04** |  | 0.12 |  | 0.12 | 0.61 |  |  |  |
| **Age at menarche** |  | |  |  |  |  |  |  |  |  |  |  |  |
| ≤12 years | 48 | | 1.0 (ref) | 69 | 1.0 (ref) | 97 | 1.0 (ref) | 86 | 1.0 (ref) | 1.0 (ref) |  |  |  |
| 13 years | 28 | | 0.49 (0.20, 1.17) | 37 | 0.84 (0.40, 1.77) | 59 | 1.20 (0.66, 2.20) | 46 | 1.25 (0.64, 2.45) | 0.58 (0.25, 1.38) |  |  |  |
| ≥14 years | 25 | | 0.68 (0.28, 1.67) | 30 | 0.62 (0.28, 1.40) | 33 | 1.33 (0.63, 2.80) | 31 | 0.89 (0.41, 1.94) | 1.03 (0.42, 2.51) |  |  |  |
| p-trend |  | 0.29 | |  | 0.26 |  | 0.41 |  | 0.94 | 0.78 | |  |  |
| **Parity** | | | |  |  |  |  |  |  |  |  | |  |
| Nulliparous | 8 | | 1.0 (ref) | 12 | 1.0 (ref) | 14 | 1.0 (ref) | 16 | 1.0 (ref) | 1.0 (ref) |  |  |  |
| 1 birth | 6 | | 2.15 (0.29, 16.2) | 8 | 4.05 (0.73, 22.3) | 14 | 0.97 (0.23, 4.04) | 9 | 1.07 (0.23, 4.95) | **17.8 (1.64, 194.3)** |  |  |  |
| ≥2 births | 48 | | 1.43 (0.36, 5.69) | 63 | 2.93 (0.87, 9.87) | 89 | 1.11 (0.34, 3.65) | 78 | 1.45 (0.50, 4.23) | 7.57 (0.91, 63.1) |  |  |  |
| p-trend |  | | 0.73 |  | 0.13 |  | 0.82 |  | 0.47 | 0.11 |  |  |  |
| **Age at first birth (among parous women)** | | | |  |  |  |  |  |  |  |  |  |  |
| <25 years | 33 | | 1.0 (ref) | 42 | 1.0 (ref) | 64 | 1.0 (ref) | 51 | 1.0 (ref) | 1.0 (ref) |  |  |  |
| 25-29 years | 16 | | 0.58 (0.25, 1.36) | 21 | 1.75 (0.83, 3.68) | 30 | 1.36 (0.72, 2.58) | 28 | 0.85 (0.43, 1.68) | 0.68 (0.30, 1.53) |  |  |  |
| ≥30 years | 5 | | 0.48 (0.14, 1.66) | 8 | 2.16 (0.71, 6.55) | 9 | 1.05 (0.40, 2.79) | 8 | 2.53 (0.80, 7.98) | 0.76 (0.23, 2.58) |  |  |  |
| p-trend |  | | 0.19 |  | 0.12 |  | 0.70 |  | 0.18 | 0.51 |  |  |  |
| **Birth Index** |  | |  |  |  |  |  |  |  |  |  |  |  |
| ≤30 | 27 | | 1.0 (ref) | 47 | 1.0 (ref) | 68 | 1.0 (ref) | 55 | 1.0 (Ref) | 1.0 (ref) |  |  |  |
| 31-59 | 35 | | 0.82 (0.32, 2.07) | 47 | 1.02 (0.47, 2.23) | 63 | 0.95 (0.48, 1.89) | 53 | 1.07 (0.51, 2.25) | 1.31 (0.53, 3.21) |  |  |  |
| ≥60 | 34 | | 0.77 (0.30, 1.98) | 36 | 0.67 (0.28, 1.59) | 50 | 1.08 (0.50, 2.31) | 48 | 0.67 (0.30, 1.49) | 0.71 (0.25, 2.03) |  |  |  |
| p-trend |  | | 0.60 |  | 0.34 |  | 0.83 |  | 0.27 | 0.41 |  |  |  |
| **Total breastfeeding (among parous women)** | | | |  |  |  |  |  |  |  |  |  |  |
| Never | 24 | | 1.0 (ref) | 28 | 1.0 (ref) | 31 | 1.0 (ref) | 36 | 1.0 (ref) | 1.0 (ref) |  |  |  |
| <6 months | 14 | | 0.43 (0.12, 1.51) | 17 | 0.70 (0.23, 2.18) | 31 | 0.71 (0.28, 1.77) | 20 | 1.35 (0.47, 3.88) | 0.59 (0.19, 1.86) |  |  |  |
| ≥6 months | 16 | | **0.18 (0.05, 0.68)** | 26 | 0.71 (0.26, 1.98) | 40 | 0.85 (0.36, 2.03) | 31 | 1.73 (0.67, 4.48) | 0.88 (0.34, 2.32) |  |  |  |
| p-trend |  | | **0.02** |  | 0.62 |  | 0.99 |  | 0.30 | 0.97 |  |  |  |
| **First-degree family history of breast cancer** | | | |  |  |  |  |  |  |  |  |  |  |
| Absent | 81 | | 1.0 (ref) | 112 | 1.0 (ref) | 156 | 1.0 (ref) | 131 | 1.0 (ref) | 1.0 (ref) |  |  |  |
| Present | 21 | | 1.60 (0.65, 3.96) | 26 | 1.75 (0.78, 3.92) | 35 | 0.95 (0.49, 1.87) | 34 | 0.82 (0.40, 1.67) | 0.91 (0.38, 2.21) |  |  |  |
| **Alcohol consumption** |  | |  |  |  |  |  |  |  |  |  |  |  |
| <0.4 drink/wk | 33 | | 1.0 (ref) | 48 | 1.0 (ref) | 70 | 1.0 (ref) | 51 | 1.0 (ref) | 1.0 (ref) |  |  |  |
| 0.4 – 2.4 drink/wk | 27 | | 1.01 (0.39, 2.61) | 44 | 1.30 (0.61, 2.76) | 62 | 1.29 (0.69, 2.43) | 52 | 1.86 (0.90, 3.86) | 0.83 (0.36, 1.91) |  |  |  |
| ≥2.5 drink/wk | 38 | | 1.92 (0.80, 4.60) | 43 | **3.11 (1.42, 6.83)** | 56 | 0.89 (0.47, 1.71) | 58 | 1.61 (0.80, 3.26) | 0.55 (0.23, 1.30) |  |  |  |
| p-trend |  | | 0.10 |  | **0.004** |  | 0.58 |  | 0.37 | 0.17 |  |  |  |
| **Physical activity** |  | |  |  |  |  |  |  |  |  |  |  |  |
| <5.5 MET-hr/wk | 31 | | 1.0 (ref) | 38 | 1.0 (ref) | 57 | 1.0 (ref) | 49 | 1.0 (ref) | 1.0 (ref) |  |  |  |
| 5.5 – 15.4 MET-hr/wk | 25 | | 0.60 (0.22, 1.61) | 42 | 0.82 (0.36, 1.84) | 61 | 0.90 (0.46, 1.77) | 46 | 2.88 (1.32, 6.29) | 1.82 (0.73, 4.55) |  |  |  |
| ≥15.5 MET-hr/wk | 34 | | 0.90 (0.37, 2.22) | 47 | 0.74 (0.34, 1.62) | 56 | 1.17 (0.59, 2.32) | 54 | 1.28 (0.63, 2.61) | 1.46 (0.60, 3.57) |  |  |  |
| p-trend |  | | 0.98 |  | 0.49 |  | 0.56 |  | 0.98 | 0.57 |  |  |  |

Note: Odds ratios and 95% confidence intervals were estimated using ordinal logistic regression models on marker expression (<7.3, 7.3-14.5, ≥14.6% ER-positive cells; <4.0, 4.0-9.9, ≥10.0% PR-positive cells; <2.3, 2.3-6.1, ≥6.2% Ki67-positive cells), adjusted for age (years). P-trend was estimated using the Wald test for continuous variables (category-specific median values).

Abbreviations: ER=estrogen receptor, PR=progesterone receptor, OR=odds ratio, CI=confidence interval, MET-hr/wk=metabolic equivalent of task-hours/week
